# Inhibition of VEGF-Induced VEGFR-2 Activation and HUVEC Migration by Melatonin and Other Bioactive Indolic Compounds

**DOI:** 10.3390/nu9030249

**Published:** 2017-03-08

**Authors:** Ana B. Cerezo, Ruth Hornedo-Ortega, M. Antonia Álvarez-Fernández, Ana M. Troncoso, M. Carmen García-Parrilla

**Affiliations:** Departamento de Nutrición y Bromatología, Toxicología y Medicina Legal, Facultad de Farmacia, Universidad de Sevilla, C/P. García González s/n, 41012 Sevilla, Spain; acerezo@us.es (A.B.C.); rhornedo@us.es (R.H.-O.); malvarez2@us.es (M.A.Á.-F.); amtroncoso@us.es (A.M.T.)

**Keywords:** melatonin, serotonin, 3-indolacetic acid, 5-hydroxytryptophol, VEGF, VEGFR-2, anti-angiogenic, HUVEC, migration

## Abstract

Excessive concentrations of vascular endothelial growth factor (VEGF) trigger angiogenesis, which causes complications such as the destabilization of atherosclerotic plaques and increased growth of tumors. This work focuses on the determination of the inhibitory activity of melatonin and other indolic related compounds on VEGF-induced VEGF receptor-2 (VEGFR-2) activation and an approximation to the molecular mechanism underlying the inhibition. Quantification of phosphorylated VEGFR-2 was measured by ELISA. Migration wound-healing assay was used to determine cell migration of human umbilical vein endothelial cells (HUVECs). This is the first time that melatonin, 3-indolacetic acid, 5-hydroxytryptophol, and serotonin are proved to significantly inhibit VEGF-induced VEGFR-2 activation in human umbilical vein endothelial cells and subsequent angiogenesis. 3-Indolacetic acid showed the highest inhibitory effect (IC_50_ value of 0.9704 mM), followed by 5-hydroxytryptophol (35% of inhibition at 0.1 mM), melatonin (30% of inhibition at 1 mM), and serotonin (24% of inhibition at 1 mM). An approximation to the molecular mechanism of the inhibition has been proposed, suggesting that indolic compounds might interact with the cell surface components of the endothelial membrane in a way that prevents VEGF from activating the receptor. Additionally, wound-healing assay revealed that exposure of HUVECs to melatonin and 3-indolacetic acid in the presence of VEGF significantly inhibited cell migration by 87% and 99%, respectively, after 24 h. These data demonstrate that melatonin, 3-indolacetic acid, 5-hydroxytryptophol, and serotonin would be good molecules for future exploitation as anti-VEGF signaling agents.

## 1. Introduction

Angiogenesis, the physiological process in which new blood vessels are formed from pre-existing ones, plays a critical role on tumor progression and development, as well as in the development and destabilization of atherosclerotic plaques [1,2]. Angiogenesis takes place when there is an imbalance between pro-angiogenic and anti-angiogenic factors. Vascular endothelial growth factor (VEGF) is the most active endogenous pro-angiogenic factor in humans [3,4,5,6]. VEGF triggers angiogenesis by binding to type III receptor tyrosine kinases on the cell surface, which causes the receptors to dimerize and become activated through transphosphorylation. VEGF receptor 2 (VEGFR-2) is the main mediator of the proliferation, migration, survival and permeability enhancing effect of VEGF [3,7,8]. Additionally, these VEGF responses can be further promoted by a small number of VEGFR-2 co-receptors such as neuropilins (NRP) or heparan sulfate proteoglycans (HSPG) [9,10]. VEGF stimulation of atherosclerotic plaque progression [1,11] and tumor angiogenesis [12] has been demonstrated. Indeed, VEGF and VEGFR-2 are molecular targets for drug therapies that aim for the inhibition of VEGF signaling [7].

Melatonin is an ubiquitous molecule, which has been found not only in the human pineal gland but also in vegetables and their fruits and seeds, medicinal herbs, and fermented products such as nuts, tomatoes, beetroots, cucumber, banana, strawberry, cherry, apple, walnut, pistachio, bread, cocoa powder, green coffee, mustard seeds, feverfew, St John’s Wort, olive oil, wine, beer, etc., at concentrations varying between 5 pg/g or mL to 230 µg/g or mL [13,14,15,16,17,18,19,20,21,22,23,24,25,26,27,28,29]. According to the 2011 EFSA Comprehensive European Food Consumption Database (France, Germany, United Kingdom, Spain and Italy) [30], the mean intake of melatonin from the consumption of just twelve food items (tomato, beetroot, banana, strawberry, cherry, apple, walnut, pistachio, bread, olive oil, wine, and beer) ranges between 4 and 550 µg/day, with the United Kingdom and Spain being the countries with the lowest and highest intake of melatonin, respectively. Considering melatonin bioavailability (up to 56%, with a mean value of 19%) [31,32,33,34] and plasma volume (5 L), the circulating melatonin would be between 0.15 and 21 ng/mL (for mean melatonin bioavailability = 19%). It is worth mentioning that melatonin concentration in human serum is 10–30 pg/mL [18,20]. These data reveal that melatonin intake could be between 15 and 700 fold higher than endogenous melatonin. In fact, it has been observed that a moderate intake of beer (330–660 mL) significantly increased melatonin concentration in plasma [20]. Moreover, melatonin supplements are widely commercialized in Europe [35]. The European Union considers food supplements as food, and thus they are regulated by European food law [36,37]. Melatonin content in supplements vary between 1 and 1.95 mg/unit. It should be noticed that melatonin bears two health claims already authorized by the EU Commission [38].

Melatonin has received a great deal of attention not only because it synchronizes circadian rhythms [39,40] but also because of its potential use to prevent and treat cardiovascular diseases and some types of cancer [41,42,43,44,45,46,47]. Melatonin has proved to be an antiangiogenic molecule [41,48]. On the one hand, melatonin has shown to inhibit angiogenesis indirectly by down-regulating VEGF expression and secretion in tumor cells [49,50]. In fact, melatonin has been proved to decrease VEGF blood levels in advanced cancer patients [41]. Previous studies support that melatonin inhibits VEGF expression by reducing HIF-1α expression or protein concentration in tumor cells [49,51,52,53,54,55,56]. On the other hand, melatonin has been proposed to directly inhibit angiogenesis by inhibiting endothelial cell proliferation via: (i) modulation of P53 and Bax/Bcl-2 expression, blocking the cell cycle and inducing cellular apoptosis respectively; and (ii) modulation of melatonin receptors/ERK1/2/PI3K/AKT/PKC/NF-ĸB pathways [57,58]. It should be noted that the latter set of signaling kinases are also modulated via VEGFR-2 signaling activation by VEGF on human umbilical vein endothelial cells (HUVECs) [8]. However, the mechanism of action by which melatonin exerts its direct anti-angiogenic effect on endothelial cells in the presence of VEGF, which is the most active endogenous pro-angiogenic factor, while considering the activation of VEGFR-2, the main mediator of the proliferation, migration, survival, and permeability of endothelial cells [3,7,8], is still unexplored. 

Tryptophan is the essential precursor molecule for melatonin synthesis in animals and plants [18]. In addition to melatonin, other related indolic compounds derived from tryptophan metabolism, such as serotonin, 5-hydroxytryptophol, and 3-indolacetic acid [59,60,61], have also been found to possess anti-angiogenic, anti-proliferative, and pro-apoptotic effects on endothelial and tumor cells [59,62,63]. However, their ability to inhibit VEGF-mediated angiogenesis has not been studied yet. Serotonin and 5-hydroxytryptophol are pineal indoles [59,60]. Furthermore, serotonin is produced in wine and beer by yeast during fermentation at concentrations of 2–24 µg/mL [24,64,65]. 3-Indolacetic acid is a colonic tryptophan metabolite [60], which is also synthesized by yeast during wine fermentation at levels of up to 500 ng/mL [64,65].

The original aim of the present study was to determine the inhibitory effect of melatonin, serotonin, 5-hydroxytryptophol, and 3-indolacetic acid on VEGF-induced VEGFR-2 activation in human umbilical vein endothelial cells as a direct mechanism of action of angiogenesis inhibition by indolic compounds and to provide insight into the possible molecular mechanism. 

## 2. Materials and Methods

### 2.1. Cell Culture

Human umbilical vein endothelial cells (HUVECs) were obtained from Lonza (Slough, UK). They were maintained in Endothelial Cell Growth Medium-2 (EGM-2) (Lonza, Slough, UK). The cells were cultured at 37 °C in an atmosphere at 5% CO_2_. All experiments were performed with HUVECs between passages 4 and 5.

### 2.2. Study Compounds

Melatonin, serotonin, and 3-indolacetic acid were purchased from Sigma-Aldrich (St. Louis, MO, USA). 5-hydroxytryptophol was obtained from Cayman Chemical (Ann Arbor, MI, USA). 

### 2.3. Treatments of HUVECs

Confluent HUVECs were washed two times with warm phosphate-buffered saline (PBS) before the addition of either vehicle controls (≤0.1% DMSO) or indolic compounds (melatonin, serotonin, 3-indolacetic acid, or 5-hydroxytryptophol at final concentration of ≤0.1% DMSO) at 0.001–1 mM concentrations using endothelial basal medium (EBM) (EGM-2 with no serum or growth factors, only including antibiotics). The treatments were 4 and 24 h of incubation, with and without a washing step, prior to stimulation with 25 ng/mL VEGF (human recombinant VEGF_165_, R&D Systems, Minneapolis, MN, USA) for 5 min. After the treatments, the cells were lysed with radioimmunoprecipitation assay buffer containing protease and phosphatase inhibitors (Roche Molecular Biochemical, Barcelona, Spain). The protein content of the lysates was determined through bicinchoninic acid assay (St. Louis, MO, USA). For IC_50_ value determination, five different concentrations were tested (0.1–2.5 mM) at 4 h of incubation.

Moreover, following the procedure in Moyle et al. [66], melatonin and 3-indoleacetic acid were pre-incubated with VEGF before being exposured to HUVECs. Briefly, 0.1 and 1 mM melatonin and 3-indoleacetic acid (stocks dissolved in DMSO) were incubated with VEGF (25 ng/mL) in endothelial basal medium for 5 min (final concentration of ≤0.1% DMSO). Confluent HUVECs were washed two times with warm PBS prior to the addition of the mixture of VEGF and melatonin for 5 min. Control incubations included a vehicle control (equivalent concentration of DMSO, ≤0.1%) and VEGF (25 ng/mL and final concentration of ≤0.1% DMSO) alone. The cells were lysed and the protein content was determined as mentioned above. 

### 2.4. Phosphorylated VEGFR-2 ELISA

Phosphorylated VEGFR-2 in the lysates was quantified using a PathScan Phospho-VEGFR-2 (Tyr1175) sandwich ELISA kit (Cell Signaling Technology, Danvers, MA, USA), following the manufacturer’s instructions.

### 2.5. Western Blot Analysis for VEGR-2

The protein lysates were mixed with NuPAGE lithium dodecyl sulfate (LDS) sample buffer, NuPAGE DTT (Invitrogen, Loughborough, UK), and denatured by heating at 70 °C for 10 min. The protein contents were then subjected to electrophoresis on NuPAGE 4%–12% Bis-Tris gels (Invitrogen) before being transferred to 0.2 μm nitrocellulose membranes (Bio-Rad, Hercules, CA, USA). Membranes were blocked with 5% bovine serum albumin (BSA) in Tris-buffered saline with Tween 20 (TBST) buffer and incubated overnight at 4 °C with antibodies directed against phospho-VEGFR-2 (Tyr 1175) and VEGFR-2 (Cell Signaling Technology). The membranes were then incubated for 1h at room temperature with anti-rabbit IgG-HRP antibodies (Cell signaling Technology) in 5% bovine serum albumin (BSA) in tris buffered saline with Tween^®^ 20 (TBST). The immunoreactive bands were detected using SuperSignal West Pico chemiluminescent substrate (Thermo Scientific, Hitchin, UK) and visualised on an Amersham Imager 600 station (GE Healthcare live sciences, Marlborough, MA, USA).

### 2.6. Migration Wound-Healing Assay

HUVECs were seeded onto 50 mm imaging dishes (Ibidi, Martinsried, Germany). When the cells achieved 100% confluence, a straight lesion was made in the center of the monolayer using a sterile 200-μL pipette tip. The wells were subsequently washed twice with PBS to remove the dead cells and incubated with EBM, containing 1 mM melatonin (final concentration of ≤0.1% DMSO), 1 mM 3-indolacetic acid (final concentration of ≤0.1% DMSO), or vehicle controls (≤0.1% DMSO) for 4 h before being treated with VEGF (25 ng/mL) for 24 h. The wounds were photographed using phase contrast microscopy on an inverted microscope (Nikon, Tokyo, Japan). Initial and final wound sizes were determined using the Nis-Elements BR v.4.30.02 software (Nikon, Tokyo, Japan), and the difference was used to determine the migration distance using the following formula; initial wound size minus final wound size divided by two. Three independent experiments were carried out. 

### 2.7. Statistical Analysis

Statistical analyses were carried out using Graphpad Prism software (GraphPad Software, Inc., San Diego, CA, USA). Student’s *t* test and one-way ANOVA (Dunnett’s multiple comparisons test) were used to test significant differences between samples.

## 3. Results

### 3.1. Inhibition of VEGF-Induced VEGFR-2 Activation by Melatonin

**Figure 1:**
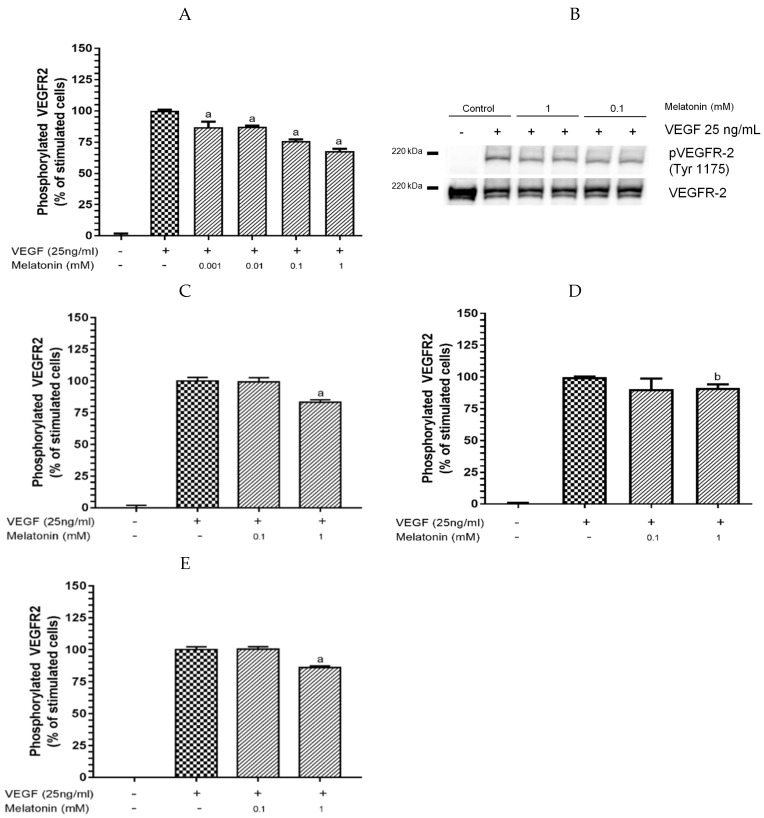
Melatonin inhibits vascular endothelial growth factor (VEGF)-induced VEGF receptor 2 (VEGFR-2) activation. Human umbilical vein endothelial cells (HUVECs) were incubated with: (**A**,**B**) different melatonin concentrations (0.001–1 mM) for 4 h; (**C**) 0.1 and 1 mM melatonin for 4 h with a subsequent washing step with phosphate-buffered saline (PBS); and (**E**) 0.1 and 1 mM melatonin for 24 h, before stimulation with VEGF (25 ng/mL) for 5 min; and (**D**) 0.1 and 1 mM melatonin were incubated with VEGF (25 ng/mL) for 5 min before exposure to HUVECs for 5 min. Phosphorylated VEGFR-2 was determined by ELISA (**A**,**C**,**D**,**E**). Data are expressed as mean ± standard deviation (SD) (*n* = 4). ^a^ *p* < 0.0001; ^b^ *p* < 0.05 versus stimulated cells. (**B**) Western blot *n* = 4.

**Figure 2:**
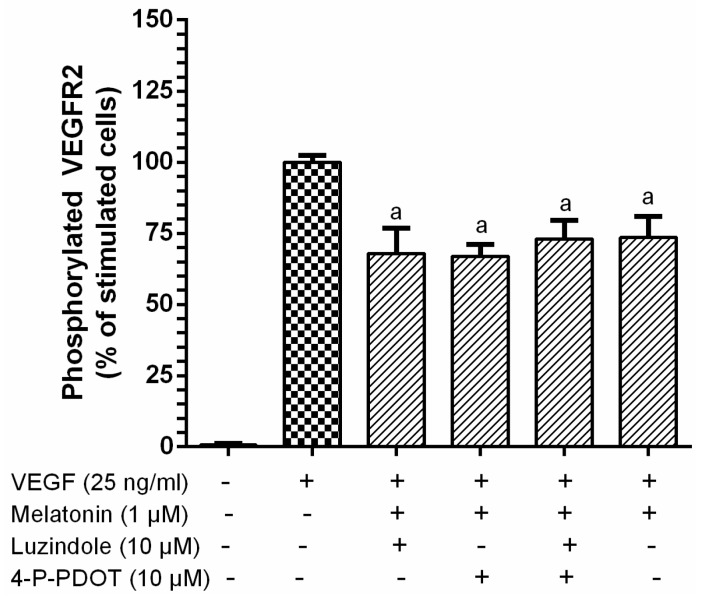
Melatonin receptors, MT1 and MT2, are not involved in the inhibition of VEGF-induced VEGFR-2 activation by melatonin. HUVECs were incubated with luzindole and 4-P-PDOT at 10 µM for 2 h and subsequently incubated with melatonin at 1 mM for 4 h, before stimulation with VEGF (25 ng/mL) for 5 min. Phosphorylated VEGFR-2 was determined by ELISA. Data are expressed as mean ± SD (*n* = 4). ^a^ *p* < 0.0001 compared to the stimulated cells.

Five different experiments were designed to explore the mechanism of action resulting in the inhibition of VEGF-induced VEGFR-2 activation by melatonin (Figure 1 and Figure 2). The first experiment involved pre-incubation of melatonin at different concentrations (0.001–1 mM) or vehicle control with HUVECs for 4 h prior to stimulation with VEGF (25 ng/mL) for 5 min. Figure 1A shows that HUVECs stimulated only with VEGF resulted in significant increases in VEGFR-2 phosphorylation. However, the pre-incubation treatment with melatonin significantly decreased VEGFR-2 phosphorylation in a dose-dependent manner from 0.01 to 1 mM. The higher the concentration is, the higher the inhibitory effect. Melatonin inhibited VEGF-induced VEGFR-2 activation by 24% and 32% at 0.1 and 1 mM concentrations, respectively, without affecting the total protein content of VEGFR-2 (Figure 1B, Table 1). The results of this experiment support the notion that melatonin is interacting with any of the following components and molecules to significantly inhibit VEGF-induced VEGFR-2 activation; (i) VEGFR-2 or any of its co-receptors such as neuropilins (NRP) or heparan sulfate proteoglycans (HSPG) at the extracellular domain; (ii) any sub-cellular kinase; or (iii) a VEGF molecule.

Secondly, we incubated HUVECs with melatonin, at the concentrations which had been proved to have a higher inhibitory effect (0.1 and 1 mM), or with vehicle control for 4 h. Subsequently, the cells were rinsed twice with PBS, and new media containing only VEGF (25 ng/mL) was added for 5 min. Figure 1C shows that only 1 mM melatonin was effective at significantly reducing VEGF-induced VEGFR-2 activation (17% of inhibition), although the inhibition percentage was ~2-fold lower compared to the conditions described for the first experiment (Figure 1A). These data indicate that melatonin should be mainly interacting with the cell surface components of the endothelial membrane, probably by a non-strong binding, which can be lost in the washing step. Additionally, interaction of melatonin with any sub-cellular kinase might occur, which would explain the 17% of melatonin inhibition at 1 mM.

The third experimental design consisted of pre-mixing melatonin with VEGF prior to being exposed to HUVECs to determine whether melatonin binds directly to VEGF molecules, as occurs with other bioactive compounds such as polyphenols [66,67]. Melatonin (at 0.1 and 1 mM) was incubated with VEGF (25 ng/mL) for 5 min prior to being added to HUVECs for 5 min. Only 1 mM melatonin showed a significant decrease of VEGFR-2 phosphorylation (8%) (Figure 1D). This percentage of inhibition was substantially lower compared to the inhibitory effect exerted by melatonin under the conditions described for the first experimental design (32%) (Figure 1A). Thus, this observation reveals that the inhibitory effect of melatonin is not likely to be mediated by the direct binding of melatonin to VEGF molecules.

Since pre-incubation of melatonin with HUVEC for 4 h and subsequent stimulation with VEGF for 5 min were the most effective conditions for the inhibition of VEGF-induced VEGFR-2 activation, we extended the pre-incubation time to 24 h to check whether the inhibitory effect was time-dependent. However, 0.1 mM melatonin did not show inhibition, and 1 mM melatonin showed only 14% of inhibition (Figure 1E), which is two times lower compared to the results of the 4 h pre-incubation experiment (Table 1). These data demonstrate that a period of 24 h does not improve the inhibitory effect of melatonin.

Melatonin membrane receptors MT1 and MT2 have been described to be involved in mediating melatonin antiproliferative properties in HUVECs; melatonin binds to these receptors and inhibits the activation of the intracellular cascade ERK/PI3K/Akt/PKC/ NF-κB [57]. To exclude melatonin receptor-mediated effects on the inhibition of VEGF-induced VEGFR-2 activation, melatonin receptors MT1 and MT2 were blocked using special antagonists, luzindole and 4-P-PDOT, and the inhibitory effect of melatonin on VEGF stimulation was measured. The results show that there are no significant differences in melatonin phospho-VEGFER-2 inhibitory effect compared with the luzindole and 4-P-PDOT treatments in the presence of melatonin and VEGF under the same conditions (Figure 2). Thus, MT1 and MT2 receptors are not involved in the inhibitory effect of melatonin on the VEGFR-2 activation of VEGF.

All the results that originated from the abovementioned experiments support the notion that the interaction of melatonin with the cell surface components of the endothelial membrane, in a way that prevents VEGF from activating the receptor, might be the dominant mechanism, resulting in the highest inhibition of VEGF-induced VEGFR-2 activation by melatonin.

### 3.2. Inhibitory Effect of Other Indolic Related Compounds on VEGF-Induced VEGR-2 Activation

We also evaluated the inhibitory effect of other related indolic compounds derived from tryptophan metabolism, such as 3-indolacetic acid, 5-hydroxytryptophol, and serotonin, on VEGF-induced VEGR-2 activation and whether they share the same mechanism of action. For comparative purposes, these compounds were tested following the same experimental conditions and concentrations (0.1 and 1 mM) mentioned above for melatonin.

**Figure 3:**
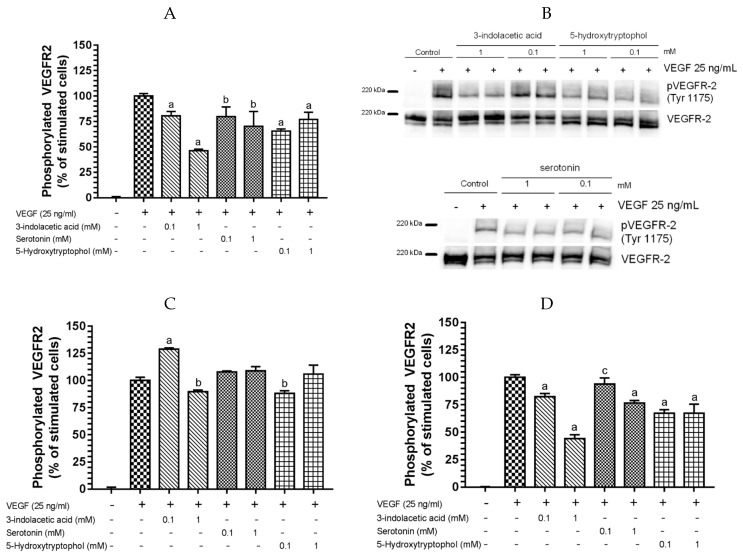

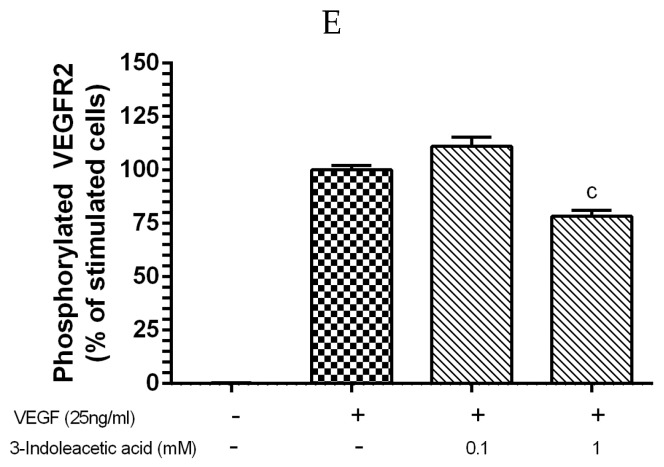
Indolic compounds inhibit VEGF-induced VEGFR-2 phosphorilation. HUVECs were incubated with 0.1 and 1 mM 3-indolacetic acid, 5-hydroxytryptophol, or serotonin for (**A**,**B**,**C**) 4 h and (**D**) 24 h before stimulation with VEGF (25 ng/mL) for 5 min. Additionally, (**C**) a washing step with PBS was included just before stimulation with VEGF (25 ng/mL); (**E**) 0.1 and 1 mM 3-indoleacetic acid were incubated with VEGF (25 ng/mL) for 5 min before exposure to HUVECs for 5 min. Phosphorylated VEGFR-2 was determined by ELISA (**A**,**C**,**D**,**E**). Data are expressed as mean ± SD (*n* = 4). ^a^ *p* < 0.0001; ^b^ *p* < 0.001; ^c^ *p* < 0.05 compared to the stimulated cells. (**B**) Western blot *n* = 4.

Figure 3 shows that the inhibition of VEGF-induced VEGR-2 activation by 3-indolacetic acid, serotonin and 5-hydroxytryptophol follow a trend similar to that of melatonin. First, the pre-incubation treatment with the indolic compounds for 4 h prior to stimulation with VEGF significantly decreased VEGFR-2 phosphorylation for all the compounds and concentrations tested (Figure 3A), without affecting VEGFR-2 total protein (Figure 3B). The higher the concentration, the higher the inhibitory effect for 3-indolacetic acid and serotonin. Conversely, the lowest concentration tested for 5-hydroxytryptophol (0.1 mM) exerted the highest inhibitory activity for this compound (Figure 3A). 3-Indolacetic acid showed the highest inhibitory effect (54% of inhibition at 1 mM), followed by 5-hydroxytryptophol (34% of inhibition at 0.1 mM) and serotonin (29% of inhibition at 1 mM). Additionally, 50% inhibitory efficacy was determined for 3-indoleacetic acid, the most effective indole, which showed an IC_50_ value of 0.9704 mM (Table 1).

Second, the pre-incubation treatment with the indolic compounds for 4 h, including a washing step before stimulation with VEGF, showed that only 1 mM 3-indolacetic acid and 0.1 mM 5-hydroxytryptophol were effective at significantly reducing VEGF-induced VEGFR-2 activation (10% and 12% of inhibition, respectively) (Figure 3C, Table 1). Moreover, the reductions in the inhibition percentages was five and three times lower for 1 mM 3-indolacetic acid and 0.1 mM 5-hydroxytryptophol, respectively, compared to the conditions described for the first experimental design (Figure 3A,C).

In addition, 0.1 and 1 mM 3-indolacetic acid and 5-hydroxytryptophol and 1 mM serotonin showed similar inhibition percentages at 24 h (Figure 3D, Table 1) than at 4 h of pre-incubation treatment (Figure 3A). However, 0.1 mM serotonin showed 6% inhibition, which is >3 times lower compared to the results of the 4 h pre-incubation experiment.

Finally, the pre-mixing experiment of 0.1 and 1 mM 3-indolacetic acid, the most effective indole, with VEGF prior to exposure to HUVECs showed that only 1 mM 3-indoleacetic acid was effective in reducing VEGFR-2 activation (22% of inhibition) (Figure 3E). However, the inhibition percentage was 2.5 times lower compared to the results of the first experiment (54% of inhibition).

These data reinforce the putative mechanism proposed above for melatonin and support that the dominant mechanism resulting in the highest inhibition of VEGFR-2 activation activity of VEGF by indolic compounds might be the interaction of indolic compounds with the surface components of the endothelial cell membrane in a way that prevents VEGF from activating the receptor.

### 3.3. Effects of Melatonin and 3-Indolacetic Acid on HUVECs Migration

**Figure 4:**
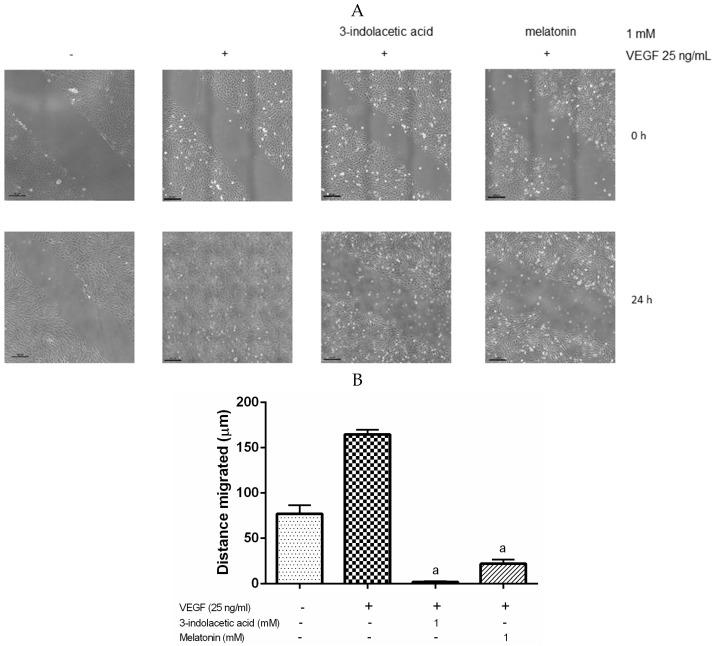
Melatonin and 3-indolacetic acid inhibit cell migration. HUVECs were wounded and incubated with melatonin or 3-indolacetic (1mM) for 4 h prior to stimulation with VEGF (25 ng/mL). (**A**) Representative photomicrographs at the beginning and after 24 h of VEGF stimulation are shown; (**B**) The migration distance was measured as detailed in the Materials and Methods section. The data of distance migrated are expressed as mean ± SD of the three experiments. Scale bars: 200 µm. ^a^ *p* < 0.0001 vs. control.

Melatonin and 3-indolacetic acid, which were two of the most effective inhibitors of VEGF-mediated VEGFR-2 activation, were selected to evaluate whether they are capable of inhibiting the cell migration of HUVECs in the presence of VEGF (25 ng/mL) by wound-healing assay. After stimulation of HUVECs with VEGF (25 ng/mL) for 24 h, the wound was no longer visible and had healed completely (Figure 4). However, the pre-incubation treatment with melatonin and 3-indolacetic acid (1 mM) for 4 h prior to stimulation with VEGF significantly inhibited the distance migrated by HUVEC by 87% and 99%, respectively, after 24 h compared to the untreated cells (Figure 4).

## 4. Discussion

This is the first time that melatonin and other related indolic compounds derived from tryptophan metabolism (3-indolacetic acid, serotonin, and 5-hydroxytryptophol) are proved to inhibit VEGF-induced VEGFR-2 activation and subsequent angiogenesis. Additionally, we propose that the dominant mechanism resulting in the highest inhibition of VEGF-induced VEGFR-2 activation by indolic compounds might be the interaction of indolic compounds with the surface components of the endothelial cell membrane, without the involvement of melatonin MT1 and MT2 receptors. Additionally, we proved that melatonin and 3-indolacetic acid inhibited HUVEC cell migration in the presence of VEGF. Thus, the results of the present study provide new evidence of the anti-angiogenic mechanism of the action of melatonin and other related indolic compounds on HUVEC cells.

The ability of melatonin to inhibit the angiogenic effect on endothelial cells has been previously described [48,49,57]. Álvarez-García et al. [48] showed that melatonin reduced endothelial cell proliferation, invasion, migration, and tube formation on HUVECs, although the mechanism of action was not proved. On the other hand, two mechanisms of action have been proposed for melatonin inhibition of HUVEC proliferation; (i) modulation of P53 and Bax/Bcl-2 expression and (ii) modulation of melatonin receptors/ERK1/2/PI3K/AKT/PKC/NF-κB [57,58]. These theories were based on melatonin incubation with HUVEC for an extended period of time (24 h), and the presence of VEGF was not considered. However, our model proved that melatonin significantly inhibits VEGFR-2 phosphorylation (30%), which is the main mediator of proliferation, migration, survival, and permeability on endothelial cells [8], and the subsequent HUVEC migration (87%) at 4 h in the presence of the most active endogenous pro-angiogenic factor, VEGF. Furthermore, the results show that melatonin receptors (MT1 and MT2) are not involved in the melatonin inhibition of VEGF-induced VEGFR-2 activation (Figure 2). Additionally, it must be noted that the abovementioned set of signaling kinases (ERK1/2/PI3K/AKT/PKC/NF-κB), previously identified as a melatonin inhibition pathway for HUVEC proliferation, are also modulated via VEGFR-2 signaling activation by VEGF on HUVEC [8,57]. However, VEGFR-2 specific intracellular signaling cascades have been highlighted as triggers of proliferation, migration, survival, and increased permeability in endothelial cells, each of which contributes to the angiogenic response [8]. The data presented in this report support the notion that the putative mechanism proposed here for the first time, which suggests an interaction between melatonin and the surface components of the endothelial cell membrane in a way that prevents VEGF from activating the receptor, could be postulated as an additional mechanism for melatonin’s direct anti-angiogenic effect on endothelial cells. However, since VEGF, in addition to binding to its receptor VEGFR-2 [8], interacts with a small number of VEGFR2 co-receptors such as neuropilins or heparan sulfate proteoglycans [9,10] to promote VEGF responses, further research using NRP antagonists [10], HSPG-deficient mutant cells [68], and labelled VEGF should be conducted to provide evidence regarding the interaction and binding of indolic compounds with the specific molecular target.

3-Indolacetic acid was the most effective inhibitor of VEGF-induced VEGR-2 activation (IC_50_ value of 970.4 µM), followed by 5-hydroxytryptophol (35% of inhibition at 0.1 mM), melatonin (30% at 1 mM), and serotonin (24% of inhibition at 1 mM). A previous report has shown that polyphenol compounds inhibit VEGF-induced VEGFR-2 activation at lower concentrations, showing higher inhibition potency (IC_50_ values: 0.088–214.7 µM) [67]. However, bioavailability differs greatly between both families of compounds (up to 12% for polyphenols and up to 56% for melatonin) [31,32,33,34,69], together with the extensive metabolism undergone by polyphenol compounds. The concentrations of melatonin found in vivo (up to 239 pg/mL = 1 nM) [70] and estimated to be achievable after consumption of melatonin-rich foods (up to 21 ng/mL = 90 nM, see Introduction section) are >4 orders of magnitude lower than the IC_50_ value for the strongest indolic inhibitor. Hence, the data presented in this study show the potential for melatonin and its derivatives to inhibit VEGFR-2 activation in vitro, although the effects are likely to be undetectable in vivo after the consumption of melatonin-rich food. However, melatonin is available as a food supplement at 1.95 mg doses [33], which could go up to 1 µM in plasma concentration (for 56% melatonin bioavailability), for which the inhibition of VEGF-induced VEGFR-2 activation is 13% (Figure 1A, Table 1).

Previous studies have shown the controversial effects of 3-indoleacetic acid on endothelial and cancer cells [62,71,72,73,74,75,76,77,78,79]. On the one hand, 3-indolacetic acid (5–1794 µM) has been related to negative implications in chronic kidney diseases by increasing the tissue factor in endothelial and vascular smooth muscle cells [72,73], inducing apoptosis of endothelial progenitor cells [74] and inhibiting the transport of the efflux pump MPR4 in human embryonic kidney cells [75]. Despite that, correlations between circulating tissue factor or endothelial progenitor cells and 3-indolacetic acid concentration in patients suffering from chronic kidney diseases were low (*r* = 0.3–0.42) [72,74]. At the same time, 3-indolacetic acid (0.5–5.7 µM) has been shown to increase cell proliferation in the renal tubular epithelial cells of healthy pigs, which could have implications in kidney regeneration [71]. On the other hand, 3-indolacetic acid (0.5–10 mM) activated by horseradish peroxidase, intense pulse light, or cytokinins has been proposed as an anticancer therapy by inducing apoptosis of tumor cells from leukemic patients [62], T24 bladder carcinoma cells [76], Pc-3 prostate cancer cells [77], human melanoma cells [78], and Hela cells [79]. However, 3-indolacetic acid alone only has an apoptotic effect in tumor cells at 10 mM [62]. The results of the present study add to this controversial effect by supporting 3-indolacetic acid as an inhibitor of angiogenesis, since it inhibits VEGF-induced VEGFR-2 activation (IC_50_ = 970.4 µM) as another alternative to target cancer cells.

The pro/anti-angiogenic effects (migration and proliferation) have been described for serotonin on endothelial cells depending on cell type and concentration (0.1 nM–1 mM) [63,80,81,82,83]. At the concentration range between 1 and 10 mM, serotonin only showed an anti-proliferative effect [63]. It is worth mentioning that these effects have been observed after long periods of time (24 h). Pakala et al. [63] highlighted that the stimulation of endothelial cell proliferation by serotonin requires prolonged incubation for at least 6 h before inducing a mitogenic response. However, the data obtained in the present study show serotonin’s inhibitory effect on VEGF-induced VEGFR-2 activation at 4 h (Figure 3A). On the other hand, serotonin has specific membrane receptors in HUVECs, which activate an identical set of signaling kinases to VEGF-induced VEGFR-2 phosphorylation, leading to angiogenesis. Only Zamani and Qua [84] compared the activation of angiogenic phosphorylation signaling of serotonin and VEGF, revealing that VEGF showed higher signaling activation. They proved that serotonin did not activate VEGFR-2; however, the serotonin phosphorylation signaling effect of serotonin was not tested in the presence of VEGF. Although serotonin could activate the angiogenic phosphorylation signaling by a parallel pathway similar to VEGF-induced VEGFR-2 activation, when serotonin is exposed to HUVEC cells in the presence of VEGF, the most active endogenous pro-angiogenic factor, VEGFR-2 activation is inhibited (24%) and, as a consequence, so is angiogenesis. Further research must been carried out on the migration and proliferation effect of serotonin on endothelial cells in the presence of VEGF before highlighting serotonin as a pro-angiogenic compound.

Existing data support both the stimulatory [85,86,87,88,89,90,91] and inhibitory [59,92,93] roles of serotonin in tumor cell proliferation. Serotonin receptors have been postulated to react in a tissue-specific manner to explain the dual role of serotonin in cancer cells [94,95,96]. Serotonin concentrations might also explain its double role since, at high serotonin dosages (100 µM to 10 mM), only the inhibitory effect was observed [92]. On the other hand, serotonin (100 nM–10 mM) has been suggested to inhibit the growth of human tumors by selectively constricting tumor arterioles [92]. Indeed, inhibition of VEGF-induced VEGFR-2 activation leads to the inhibition of endothelial nitric oxide synthase (eNOS), which could explain this constricting effect of serotonin on arterioles. Further research should be conducted on the effect of serotonin on the inhibition of the intracellular signaling of VEGF-induced VEGFR-2 activation.

5-Hydroxytryptophol has been previously related to the antiproliferative effect on cultured tumor cell lines such as sarcoma, choriocarcinoma, and macrophage-like cell lines [59]. However, this is the first time that an anti-angiogenic effect, related to the inhibition of VEGF-induced VEGFR-2 activation, has been shown by 5-hydroxytryptophol on endothelial cells. Thus, further research should be carried out on the effect of this pineal indole on the proliferation and migration of endothelial cells for its possible use as an anti-angiogenic molecule. 

Since endothelial cell migration is essential for the formation of new blood vessels during angiogenesis, we also evaluated the anti-angiogenic effect of melatonin and 3-indolacetic acid, two of the most effective indoles for the inhibition of VEGF-mediated VEGFR-2 activation, on HUVECs migration in the presence of VEGF, following the same experimental conditions that showed the highest inhibitory effect (pre-incubation with melatonin and 3-indolacetic acid for 4 h prior to stimulation with VEGF). Melatonin and 3-indolacetic acid (1 mM) significantly inhibited HUVEC migration by 87% and 99%, respectively, after 24 h in the presence of VEGF (Figure 4). Álvarez-García et al. [48] showed that melatonin (1 mM) inhibited the migration of HUVECs by 32% after 8 h, although no presence of VEGF was reported. Nevertheless, our model proved, for the first time, that melatonin and 3-indolacetic acid reversed the migration induced by VEGF on HUVECs and that the inhibition of VEGF-induced VEGFR-2 activation by melatonin and 3-indolacetic acid is involved in it. These results are consistent with those of Álvarez-García et al. [48], who showed that melatonin was able to inhibit the VEGF-stimulated tubular network formation (40%) formed by HUVECs, although the mechanism of action was not explored. Since the formation of capillary-like tubular structures by endothelial cells is a pivotal step in angiogenesis, studies proving the inhibitory effect of 3-indolacetic acid on the formation of capillary-like tubular structures using matrigel assay [97,98] are needed to reinforce the results presented here.

## 5. Conclusions

In conclusion, the data presented in this study show, for the first time, that indolic compounds derived from tryptophan metabolism, such as melatonin, 3-indolacetic acid, serotonin, and 5-hydroxytryptophol, inhibit VEGF-induced VEGFR-2 activation and subsequent angiogenesis. Our data presented in this report suggest that the mechanism proposed here for the first time, which suggests the interaction between indolic compounds and surface components of the endothelial cell membrane in a way that prevents VEGF from activating the receptor, could be proposed as an additional mechanism of the anti-angiogenic effect of indolic compounds.

## Figures and Tables

**Table 1:** IC_50_ and percentage of inhibition of VEGF-induced VEGFR-2 activation by indolic compounds.

Compounds	Concentrations (mM)	% Inhibition			IC_50_ (mM)
		A	B	C	
Melatonin	0.001	13.12 ± 4.47	ND	ND	ND
	0.01	12.85 ± 0.95	ND	ND	
	0.1	24.36 ± 1.57	0.68 ± 3.35	NI	
	1	32.15 ± 1.87	16.66 ± 1.9	13.86 ± 1.12	
3-Indolacetic acid	0.1	19.41 ± 4.19	NI	17.68 ± 2.99	0.9704 (0.7174–1.313)
	1	53.56 ± 1.39	10.34 ± 1.55	55.94 ± 3.64	
Serotonin	0.1	20.21 ± 9.47	NI	6.16 ± 5.66	ND
	1	29.56 ± 14.36	NI	23.33 ± 2.29	
5-hydroxytryptophol	0.1	34.37 ± 2.11	11.95 ± 2.50	32.51 ± 3.08	ND
	1	22.99 ± 7.02	NI	32.58 ± 8.09	

ND: non-determined. NI: non-inhibition. 95% confidence intervals for the IC_50_ values are shown in parentheses. HUVECs incubated with indolic compounds for (**A**,**B**) 4 h and (**C**) 24 h before stimulation with VEGF (25 ng/mL) for 5 min. (**B**) Additional washing step with PBS included just before stimulation with VEGF (25 ng/mL).
